# Who Is Not Linking to HIV Care in Tennessee — the Benefits of an Intersectional Approach

**DOI:** 10.1007/s40615-021-01023-6

**Published:** 2021-04-19

**Authors:** Leslie J. Pierce, Peter Rebeiro, Meredith Brantley, Errol L. Fields, Cathy A. Jenkins, Derek M. Griffith, Donaldson Conserve, Bryan Shepherd, Carolyn Wester, Aima A. Ahonkhai

**Affiliations:** 1 Vanderbilt Institute for Global Health, Vanderbilt University Medical Center, 2525 West End Ave, Suite 750, Nashville, TN 37203, USA; 2 Department of Medicine, Infectious Diseases, Vanderbilt University Medical Center, Nashville, TN, USA; 3 Department of Biostatistics, Vanderbilt University School of Medicine, Nashville, TN, USA; 4 Department of Medicine, Division of Epidemiology, Vanderbilt University School of Medicine, Nashville, TN, USA; 5 Tennessee Department of Health, Nashville, TN, USA; 6 Division of Adolescent/Young Adult Medicine, Department of Pediatrics, Johns Hopkins School of Medicine, Baltimore, MD, USA; 7 Center for Research on Men’s Health, Vanderbilt University, Nashville, TN, USA; 8 Department of Health Promotion, Education and Behavior, University of South Carolina, Columbia, SC, USA

**Keywords:** HIV care continuum, Structural drivers, Linkage to care, Intersectionality

## Abstract

**Introduction:**

Guided by an intersectional approach, we assessed the association between social categories (individual and combined) on time to linkage to HIV care in Tennessee.

**Methods:**

Tennessee residents diagnosed with HIV from 2012–2016 were included in the analysis (n=3750). Linkage was defined by the first CD4 or HIV RNA test date after HIV diagnosis. We used Cox proportional hazards models to assess the association of time to linkage with individual-level variables. We modeled interactions between race, age, gender, and HIV acquisition risk factor (RF), to understand how these variables jointly influence linkage to care.

**Results:**

Age, race, and gender/RF weAima A. Ahonkhaire strong individual (*p* < 0.001 for each) and joint predictors of time to linkage to HIV care (*p* < 0.001 for interaction). Older individuals were more likely to link to care (aHR comparing 40 vs. 30 years, 1.20, 95%CI 1.11–1.29). Blacks were less likely to link to care than Whites (aHR = 0.73, 95% CI: 0.67–0.79). Men who have sex with men (MSM) (aHR = 1.18, 95%CI: 1.03–1.34) and heterosexually active females (females) (aHR = 1.32, 95%CI: 1.14–1.53) were more likely to link to care than heterosexually active males. The three-way interaction between age, race, and gender/RF showed that Black males overall and young, heterosexually active Black males in particular were least likely to establish care.

**Conclusions:**

Racial disparities persist in establishing HIV care in Tennessee, but data highlighting the combined influence of age, race, gender, and sexual orientation suggest that heterosexually active Black males should be an important focus of targeted interventions for linkage to HIV care.

## Introduction

HIV does not affect all people or places in the United States (US) equally, and populations with the highest rates of HIV diagnoses often have the lowest rates of linkage to care [1]. While there is a tendency to consider demographic and place-based factors separately, an intersectional approach may provide a more nuanced way to highlight the conversion between structures and processes — as represented by race, place, age, gender, sexuality, and other factors — and the implications of such on resources and opportunities that affect health and health care [2–4]. Intersectionality highlights the connected nature of social categories and informs how intersecting categories may place socially marginalized groups at even greater risk for disease [5, 6].

First described by legal scholar Kimberle Crenshaw, intersectionality posits that an individual’s social identities (for example a young, Black, man who has sex with men, from the Southern US) are not simply the additive sum of these individual identities [7]. Rather, these identities represent larger societal structures that both co-exist and are interdependent [7]. Such perspectives have gained attention as a way to identify and contextualize those at greatest risk of acquiring HIV or having poor outcomes from HIV infection [8–11]. Intersectionality-informed approaches have highlighted how structural racism impacts HIV-related health behaviors, and how inequalities attributed to one demographic factor alone (race) do not capture fully other markers of who may be disadvantaged (such as high levels of stigma, incarceration, unemployment, and other similar sociocultural factors) which may be important for intervention development and resource allocation [8–11]. One study among Ugandan men highlighted how the intersection of masculinity and HIV-associated stigma influenced healthcare seeing behavior through their views on sickness, vulnerability, and financial responsibility; and illustrated that support group participation could be improved by focusing on income-generating activities [12]. Another study among gay Latino men in the Southern US highlighted the importance of integrating health and immigration interventions to address multilayered experiences with stigma that directly impact engagement in care and adherence to ART [13].

Race, place, age, and sexuality represent important factors that are routinely considered in describing cohorts of people living with HIV (PLWH) and identifying groups at greater risk for poor HIV outcomes. In 2017, non-Hispanic Black (Black) individuals accounted for 13% of the US population, but 43% of new HIV diagnoses nationwide [14, 15]. Black PLWH in the US also have worse HIV care and viral load outcomes relative to other races/ethnicities [14, 15]. Place has been recognized as a useful proxy for identifying populations at risk for HIV, and those who may need better access to HIV care. Southern states account for 51% of all new HIV cases despite only representing only 38% of the US population; the rates in this region may be exacerbated by structural factors like high rates of poverty, unemployment, and HIV-related stigma [16]. Furthermore, age has been shown to be an important predictor of HIV outcomes [6, 17–19]. Robust literature highlights poor HIV care outcomes for adolescents and young adults due, in part, to dynamics of this unique developmental period including impulsivity, risk-taking behavior, poor abstract thinking, nascent autonomy, and variable levels of social support [6, 17–19].

Sexual orientation is also intricately linked with HIV risk acquisition by defined behaviors and sexual networks [17, 18]. In 2017, 70% of incident HIV infections in the US were among men who have sex with men (MSM) [20]. Sexual and gender minorities, particularly those who are also racial and ethnic minorities, may contend with intersectional stigma (i.e., stigmatization based on overlapping structures defined by race, sexuality, and gender identity), isolation, and discrimination that pose substantial barriers to HIV care and treatment [15, 17, 18].

Tennessee, like the US, has witnessed persistent racial disparities in new HIV diagnoses in the state [21]. In 2017, black individuals were diagnosed with HIV at a rate of 35.9 per 100,000 persons, compared to 10.1 among Hispanic individuals and 5.2 among non-Hispanic White (White) individuals [22]. Further exacerbating this disparity, Tennessee trails the nation in linkage to HIV care and Black PLWH remain the least likely of any race/ethnicity to establish care after HIV diagnosis despite efforts by the Tennessee Department of Health (TDH) to address racial disparities in HIV outcomes [15, 22]. The objective of this analysis was to illustrate whether an intersectional approachconsidering age, race, gender, and sexual orientation from TDH surveillance data could help to better understand who has the lowest linkage to HIV care in Tennessee. Answering this question will help TDH and HIV service providers create a more tailored plan to close gaps in HIV prevention and care-related disparities, and further efforts to end the epidemic.

## Methods

We conducted a retrospective cohort analysis of Tennessee residents who were newly diagnosed with HIV between January 1, 2012, and December 31, 2016. Data were available in the same calendar year of diagnosis. We assessed individual factors associated with time from diagnosis to linkage to HIV care, defined as receipt of the first CD4 or HIV-1 RNA test result within the same calendar year as diagnosis captured via Tennessee’s enhanced HIV/AIDS reporting system (eHARS).

The individual-level variables obtained from eHARS included year of diagnosis, age at diagnosis, gender, race/ethnicity (White/non-Hispanic, Black/non-Hispanic, Hispanic/all races, other/unknown), HIV acquisition risk factor (heterosexual contact, MSM, injection drug use (IDU), MSM/IDU, other, unknown), and site of diagnosis (inpatient facility/ER, outpatient facility, health department or STD/family planning clinic, blood bank, correctional facility, other/unknown, missing). We combined gender and HIV acquisition risk factor into one indicator with the following categories: male/heterosexual, male/MSM, male/IDU, male/other-unknown, female/heterosexual, female/IDU, and female/other-unknown. Data on transgender and gender non-confirming individuals was not routinely collected by TDH for this analysis period and was thus not available for this analysis.

We used descriptive statistics (median, interquartile range [IQR] or percent, as appropriate) to summarize demographic characteristics of the cohort. We then fit Cox proportional hazards models to assess the association of time to linkage to care within the first year post diagnosis with a priori selected individual-level covariates. Year and age were included as continuous variables with age expanded using restricted cubic splines with 4 knots to relax linearity assumptions. Subjects were followed until linkage to care within the same calendar year as diagnosis, death, or end of the calendar year.

Finally, in addition to modeling main effects for all covariates, we also modeled the joint effects of age, gender/transmission risk category, and race on linkage to HIV care using a three-way interaction. Results from these models were summarized using the estimated probability of linkage to care at 30 days according to race/ethnicity, transmission risk factor, and age; other variables (year and facility of diagnosis) were held constant (2013 and inpatient facility/ER). Consistent with CDC and HIV/AIDS Bureau recommendations, linkage to care was defined at 30 days after HIV diagnosis [23]. These recommendations reflect the importance of immediate ART initiation both for individual health benefit and to decrease the risk of HIV transmission in the community, and are essential for the country’s “Ending the Epidemic” initiatives [24]. The three-way interaction analysis was restricted to Black and White individuals since they comprised over 90% of the cohort.

### IRB Approval

We obtained a waiver of consent and IRB approval from Vanderbilt University Medical Center (Protocol no. 17119, Nashville, TN, USA) and TDH (protocol no. 1097644-4).

## Results

### Description of Cohort of Tennessee Residents Newly Diagnosed with HIV

There were 3750 newly diagnosed individuals included in this analysis. The number of new HIV diagnoses gradually decreased between 2012 and 2016 (2012: 842 individuals, 2013: 756 individuals, 2014: 729 individuals, 2015: 716 individuals, 2016: 707 individuals). By the end of the study period, 207 persons (6%) died. Individuals were more likely to be men (80% vs. 20% women) and Black (59% vs. 33% White), with a median age at diagnosis of 31 years [IQR 24, 43]. Over half (55%) reported an acquisition risk factor of MSM, while 24% reported heterosexual sex and 16% risk factors that were other or unknown. Approximately one-third (34%) were diagnosed at an outpatient health facility followed by 28% at a health department or STD/family planning clinic and 20% at an inpatient facility or emergency room (Table 1). Median time to linkage to care overall was 26 days [IQR = 9, 59]. A total of 207 deaths were recorded during the analysis period. By calendar year there were 8 deaths among 100 individuals not linked to care, 4 deaths among 93, 5 deaths among 82, 12 deaths among 102, and 2 deaths among 98 individuals not linked to care (2012 to 2016 respectively).

### Individual-Level Predictors of Time to Linkage to Care

Age was a significant individual predictor of linkage to HIV care (*p* < 0.001). Younger (20 years old, adjusted hazard ratio [aHR] = 1.17, 95% confidence interval [CI]: 1.08, 1.26) and older individuals (40 and 45 years old, aHR = 1.20, and 1.29, respectively) were more likely to establish care within a shorter time compared to 30-year-olds. Blacks had significantly longer times to linkage than Whites in both unadjusted (HR = 0.70, 95% CI: 0.65–0.75) and adjusted (aHR = 0.73, 95% CI: 0.67–0.79) analyses. Time to linkage to care did not differ significantly between White and Hispanic individuals (aHR = 0.94, 95%CI: 0.80–1.11) or those with “other/unknown ethnicity” (aHR = 0.95, 95% CI: 0.78–1.17). We found that MSM (aHR = 1.18, 95% CI: 1.03–1.34) and heterosexually active females (aHR = 1.32, 95%CI: 1.14–1.53) were more likely to have a shorter time to link to care than heterosexually active males. Compared to an inpatient facility or emergency room, diagnosis at any other location was associated with a longer time to establish care (aHR = 0.71 outpatient facility, 0.52 health department or family planning clinic, 0.30 blood bank, 0.43 correctional facilities, 0.30 other locations) (Table 2).

### Three-Way Interaction Between Age, Gender/Exposure, and Race

**Fig. 1:**
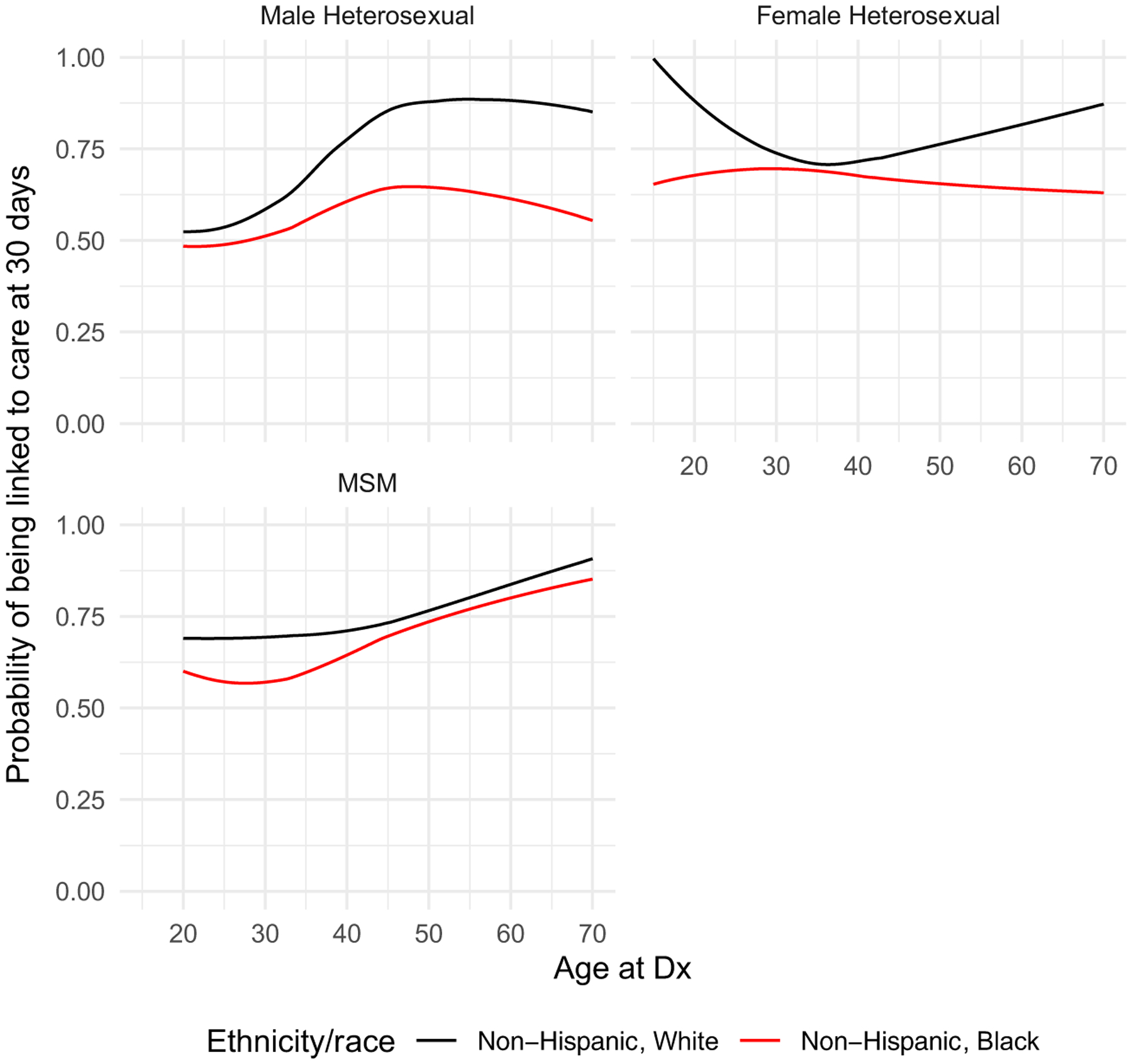
Probability of linkage to care in Tennessee. Sensitivity analysis looking at the association of time to linkage to care with a priori defined covariates, including facility type. Note that because the outcome is positive, a higher hazard indicates a shorter time to linkage to care

We modeled linkage to HIV care’s association with three-way interactions between age, gender/acquisition risk category, and race for Black and White individuals. Predicted probabilities of linkage to HIV care within 30 days of HIV diagnosis by age are illustrated in Fig. 1. In general, the three-way interactions were found to be significant (*p* < 0.001) as were the two-way individual interactions of race by gender (*p* = 0.001), race by gender/acquisition risk category (*p* = 0.001) and age by gender/acquisition risk category (*p* < 0.001). The lowest probability of linkage to HIV care at 30 days, approximately 50%, was among young, heterosexually active men. This probability was lower for Black as compared to White men. Among heterosexually active men, this racial disparity trend continued to diverge at older ages. Among MSM, racial disparities were evident among younger (< 40 years) but not older PLWH. For men, the probability of linkage increased with age. Black women had an overall consistent probability of linkage to care (62–70%) even at older ages; however, more prominent racial disparities among Black women were observed at younger (< 25 years) and older (> 50 years) ages.

## Discussion

In our cohort, while race was a strong, independent predictor of linkage to HIV care, our intersectional approach allowed us to appreciate how age, gender, and sexual orientation provided important contextualizing factors for understanding racial disparities in HIV care linkage in Tennessee. This approach underscores how looking at disparities from the perspective of race alone may obscure important, complex relationships between an individual’s social identities. Heterosexually active women were 30% more likely to link to care than heterosexually active men, and racial disparities were most notable among the youngest and oldest women in the cohort. The picture was different for men. Among heterosexually active men, the most substantial racial disparities were among those over 40 years of age. In contrast, the greatest racial disparities between Black and White MSM were observed among young men aged < 35 years. Heterosexually active Black men, however, fared worse in linking to care than any other group, at any age, and thus remain an important focus for interventions aimed at ending the epidemic.

Lower linkage to care among heterosexually active Black men, compared to Black MSM, underscores how the social identity described by sexual orientation creates a starkly different experience for Black men, and the framing of sexual orientation is an important factor in public health interventions. HIV outreach efforts and messages are often geared towards gay, bisexual, and other MSM which may alienate Black men who do not identify as gay [25]. In addition, gender and gender expression may also be an influential factor for utilization of HIV care services. Traditional masculine beliefs have been associated with poor health-seeking behaviors in both sexual minority and heterosexual Black men and research suggests that race-related threats or daily experiences of racism may further exacerbate the effect of masculinity on an individual’s attitude toward his health [26–34]. These experiences can also lead to mistrust of the healthcare system, an important contributor to negative HIV-related outcomes for Black men living with HIV [35, 36]. Utilization of nonclinical venues can potentially be leveraged as a differentiated model of care to address the concerns of these men who are often unseen by the healthcare system.

Our analysis has notable limitations. With access to historical data, we cannot comment on changes that may have occurred in linkage to care in TN between the study’s end in 2016 and the present. Additionally, our measures of linkage to care were reliant on the completeness of the mandatory reporting system, which varies by site and could have introduced some bias despite improvements in HIV surveillance and data quality since 2012. Further, we were unable to distinguish transgender individuals or those with joint MSM/IDU acquisition risk who are known to experience health disparities, and could not incorporate other important factors related to linkage to HIV, such as individual experiences with stigma, racism, and proxy measures for socio-economic status. These omissions could have introduced bias into our results.

## Conclusions

Our analysis highlights that race remains an important, independent predictor of linkage to HIV care in Tennessee and supports continued efforts to address structural racism as a driver of poor health outcomes. Furthermore, our analysis additionally highlights the importance of intersectionality in identifying populations at high risk for not establishing HIV care after diagnosis. Despite the high risk for poor linkage to care among Black PLWH in TN as a whole, we see a drastically different picture for Black women compared to Black men, and an even more contextualized picture when we consider sexuality. Our findings underscore that all Black men cannot be considered through the same lens. Without specific interventions that consider race, gender, and sexual orientation, heterosexually active Black men diagnosed with HIV in Tennessee may remain in the shadows leading to persistent disparities in HIV outcomes and undermining efforts focused on ending the epidemic in Tennessee.

## Supplementary Material

Supplemental Tables 1 and 2

## Figures and Tables

**Table 1:** Demographics of the cohort of HIV-positive individuals in Tennessee between 2012 and 2016

Demographic Value	Category	Total		30-day		60-day		90-day	
		#	%	#	%	#	%	#	%
		(*n* = 3750)		(*n* = 1840)		(*n* = 2472)		(*n* = 2747)	
Sex	Male	2987	80%	1430	78%	1921	78%	2150	78%
	Female	763	20%	410	22%	551	22%	597	22%
Race/ethnicity	White (non-Hispanic)	1230	33%	715	39%	903	37%	992	36%
	Black (non-Hispanic)	2205	59%	950	52%	1338	54%	1505	55%
	Hispanic (all races)	200	5%	109	6%	141	6%	155	6%
	Other/unknown	115	3%	66	4%	90	4%	95	3%
Age at diagnosis (years)	Median [IQR]	31	[24,43]	33	[25,45]	32	[24,45]	32	[24,45]
HIV risk factor	Heterosexual	883	24%	429	23%	595	24%	662	24%
	MSM	2079	55%	9987	54%	1371	55%	1536	56%
	IDU	114	3%	60	3%	78	3%	84	3%
	MSM/IDU	75	2%	42	2%	52	2%	56	2%
	Other/unknown	599	16%	312	17%	376	16%	409	15%
Year of diagnosis	2012	842	22%	398	22%	545	22%	617	22%
	2013	756	20%	409	22%	542	22%	580	21%
	2014	729	19%	369	20%	500	20%	551	20%
	2015	716	19%	340	18%	462	19%	516	19%
	2016	707	19%	325	18%	423	17%	483	18%
Site of diagnosis	Inpatient facility or ER	745	20%	498	27%	575	23%	610	22%
	Outpatient facility	1291	34%	717	39%	889	36%	989	36%
	Health department or STD/family planning clinic	1041	28%	360	20%	615	25%	712	26%
	Blood bank	134	4%	16	1%	39	2%	48	2%
	Correctional facility	195	5%	56	3%	94	4%	108	4%
	Other/unknown	14	0%	2	0%	5	0%	6	0%
	Missing	330	9%	191	10%	255	10%	274	10%

Demographics of people living with HIV in the state of Tennessee between 2012 and 2016

**Table 2:** Association of increased linkage to care within 30 days of HIV diagnosis with a priori defined covariates

Demographic Value	Category	Unadjusted			Adjusted		
		HR	95% CI	P	HR	95% CI	*p*
Year of diagnosis		0.97	[0.95, 0.99]	0.02	0.97	[0.95, 0.99]	0.02
Age at diagnosis (years)	20	1.15	[1.06, 1.25]	< 0.001	1.17	[1.08, 1.27]	< 0.001
	25	0.99	[0.96, 1.02]		1.02	[0.99, 1.05]	
	30 (ref)	1.00			1.00		
	40	1.32	[1.23, 1.42]		1.20	[1.11, 1.29]	
	45	1.46	[1.35, 1.58]		1.29	[1.18, 1.40]	
Sex/exposure	Male/heterosexual (ref)	1.00		< 0.001	1.00		< 0.001
	Male/MSM	1.19	[1.05, 1.35]		1.18	[1.03, 1.34]	
	Male/IDU	1.40	[1.14, 1,73]		1.20	[0.97, 1.49]	
	Male/other-unknown	1.05	[0.89, 1.23]		0.96	[0.81, 1.13]	
	Female/heterosexual	1.43	[1.23, 1.65]		1.32	[1.14, 1.53]	
	Female/IDU	1.18	[0.86, 1.62]		0.76	[0.55, 1.06]	
	Female/other-unknown	1.33	[1.09, 1.61]		0.99	[0.81, 1.20]	
Race/ethnicity	White (non-Hispanic)	1.00		< 0.001	1.00		< 0.001
	Black (non-Hispanic)	0.70	[0.65, 0.75]		0.73	[0.67, 0.79]	
	Hispanic (all races)	0.89	[0.76, 1.05]		0.94	[0.80, 1.11]	
	Other/unknown	0.96	[0.79, 1.18]		0.95	[0.78,1.17]	
Site of diagnosis	Inpatient facility or ER	1.00		< 0.001	1.00		< 0.001
	Outpatient facility	0.71	[0.64, 0.78]		0.71	[0.65, 0.79]	
	Health department or STD/family planning clinic	0.51	[0.46, 0.56]		0.52	[0.47, 0.58]	
	Blood bank	0.27	[0.21, 0.33]		0.30	[0.24, 0.37]	
	Correctional facility	0.39	[0.33, 0.47]		0.43	[0.36, 0.52]	
	Other/unknown	0.28	[0.15, 0.55]		0.30	[0.16, 0.58]	
	Missing	0.85	[0.74, 0.97]		0.83	[0.72, 0.95]	

Highest probability of failure to linkage to HIV care at 30 days was among young, heterosexual men. This shows a three-way model between age, gender/acquisition risk factor, and race
