# Supplementary material for: Who Is Not Linking to HIV Care in Tennessee — the Benefits of an Intersectional Approach
Source: J Racial Ethn Health Disparities. 2021 Apr 19;9(3):849–55. doi: 10.1007/s40615-021-01023-6 (PMC8523577; doi:10.1007/s40615-021-01023-6)
Supplement: Supplementary file 1 — (DOCX 13 kb) [file 40615_2021_1023_MOESM1_ESM.docx]

**Supplemental Table 1: Deaths among individuals not linked to care by enrollment year**

| Enrollment Year | 2012 | 2013 | 2014 | *20*15 | 2016 |
| --- | --- | --- | --- | --- | --- |
| Total Patients Enrolled | 842 | 756 | 729 | 716 | 707 |
| No. not linked to care (in calendar year) | 100 | 93 | 82 | 102 | 98 |
| Deaths among individuals not linked to care | 8 | 4 | 5 | 12 | 2 |
