# Supplementary material for: Who Is Not Linking to HIV Care in Tennessee — the Benefits of an Intersectional Approach
Source: J Racial Ethn Health Disparities. 2021 Apr 19;9(3):849–55. doi: 10.1007/s40615-021-01023-6 (PMC8523577; doi:10.1007/s40615-021-01023-6)
Supplement: Supplementary file 2 — (DOCX 15 kb) [file 40615_2021_1023_MOESM2_ESM.docx]

**Supplemental Table 2: Median time to linkage to care by baseline demographic factors**

|  | **Median** | **IQR** |
| --- | --- | --- |
| **Enrollment Year**  2012  2013  2014  2015  2016 | 28  22  25  27  28 | 9,64  7,46  9,54  10,59  10,76 |
| **Sex**  Male  Female | 26  25 | 8,62  10.50 |
| **Age Category**  <15  15-24  25-34  35-44  45-54  >54 | 1.5  32  27  23  20  15 | 0,12  16,76  12,62  6,49  3,50  1,45 |
| **HIV Risk Factor**  Heterosexual  MSM  IDU  MSM/IDU  Other/Unknown | 28  27  24  21  15 | 11,58  11,62  7,53  9,73  0,53 |
| **Race/Ethnicity**  Non-Hispanic White  Non-Hispanic Black  Hispanic/Other | 21  29  23 | 5,46  12,68  7,49 |
| **Diagnosis Facility**  Inpatient or ER  Outpatient  Health Department/STI Clinic  Blood Bank  Correctional Facility  Other/Unknown  Missing | 6  22  38  71  41  50  21 | 0,32  8, 57  21,74  39,142  20,97  33,91  3,42 |
