# Supplementary material for: Who Is Not Linking to HIV Care in Tennessee — the Benefits of an Intersectional Approach
Source: J Racial Ethn Health Disparities. Author manuscript; Available in PMC 2022 Jun 1. (PMC8523577; doi:10.1007/s40615-021-01023-6)
Supplement: Supplementary file 1 [file NIHMS1706563-supplement-Supplemental_Tables_1_and_2.pdf]

Supplemental Table 1: Deaths among individuals not linked to care by enrollment year

Enrollment Year	2012	2013	2014	*2015*	2016
Total Patients Enrolled	842	756	729	716	707
No. not linked to care (in calendar year)	100	93	82	102	98
Deaths among individuals not linked to care	8	4	5	12	2

Supplemental Table 2: Median time to linkage to care by baseline demographic factors

	**Median**	**IQR**
**Enrollment Year**		
2012	28	9,64
2013	22	7,46
2014	25	9,54
2015	27	10,59
2016	28	10,76
**Sex**		
Male	26	8,62
Female	25	10.50
**Age Category**		
<15	1.5	0,12
15-24	32	16,76
25-34	27	12,62
35-44	23	6,49
45-54	20	3,50
>54	15	1,45
**HIV Risk Factor**		
Heterosexual	28	11,58
MSM	27	11,62
IDU	24	7,53
MSM/IDU	21	9,73
Other/Unknown	15	0,53
**Race/Ethnicity**		
Non-Hispanic White	21	5,46
Non-Hispanic Black	29	12,68
Hispanic/Other	23	7,49
**Diagnosis Facility**		
Inpatient or ER	6	0,32
Outpatient	22	8, 57
Health Department/STI Clinic	38	21,74
Blood Bank	71	39,142
Correctional Facility	41	20,97
Other/Unknown	50	33,91
Missing	21	3,42
